# Bennett Park and the New Emergency Hospital

**Published:** 1899-12

**Authors:** 


### BENNETT PARK AND THE NEW EMERGENCY HOSPITAL.

AN EX-COUNCILMAN of the City of Buffalo, an old-time resident, and a man who by reason of his business associations one would naturally suppose to be broad-minded and liberal in the view he takes of public matters, has written a communication to the Board of Park Commissioners protesting against the use of public parks as breathing spots for convalescents from a projected hospital.

This gentleman is Mathias Strauss, a sheepskin tanner, a kindly-faced man, whose career in the board of councilmen was not marked by any particularly narrow-minded act; in fact, he finished out his term, as it was expected he would, as an inoffensive representative of the business interests of Buffalo, and this remarkable communication which has brought him into prominence, is really the first thing he has ever done, so far as the JOURNAL knows, which is in the slightest degree illiberal or narrow-minded.

In his communication, Mr. Strauss called the attention of the Park Commissioners to the fact that the Sisters of Charity were to build a new emergency hospital adjacent to Bennett Park, to replace the present hospital building, and he very seriously asked the commissioners to take action now to prevent the patients of this institution from frequenting the park. He said the park was now frequented by old people and children and it was undesirable to have the sick and maimed thrust before them while they were taking recreation.

This remarkable communication created considerable discussion among the members of the Park Board, who are somewhat divided on the subject. The majority of the board, the JOURNAL is glad to be able to say is made up of progressive men, who are not in any way narrow-minded or bigoted. They believe if the fresh air of the park system is for all persons of whatever condition, and that Mr. Strauss's objection is not a good one, because if any of the inmates of the hospital did occupy the park at any time, they would be convalescents who would be benefited by the air and exercise, and be not at all objectionable to other frequenters.

Yet, it strikes the JOURNAL that Mr. Strauss has arisen before the bell has rung, inasmuch as he has entered a protest against something which is not likely to happen. Ground has not yet been broken for the hospital.

As a public-spirited citizen, and a man who is disinterestedly battling for the comfort of the public at large, the JOURNAL would suggest to Mr. Strauss, that he investigate and report on the stink of tanneries and skin warehouses—stinks which are distinctly offensive and injurious to the health of neighborhoods.

The stand taken by Mr. Strauss in this matter created considerable comment and the latter wrote the following letter to an afternoon newspaper, which the JOURNAL publishes, merely as an exhibit:

I herewith wish to state and prove that Mathias Strauss is not against poor or crippled people, as stated in all the Buffalo newspapers, which I will prove at once: First, I am a member of all the Catholic institutions in this city. No man of Buffalo visits them more or helps them more when in need than M. Strauss. I am a friend of the Sisters of Charity, and have been ever since they were established in Buffalo. My whole family and myself belong to their beneficiary society, and I generally visit this institute every other week. To show that I am home with the sisters, I furnished one of the best rooms in the new wing and my sons furnished another smaller one. Within the last four weeks I have ordered two memorial-painted windows for their chapel. All this will prove that I am a friend of that institute. The reason I oppose is that I know the financial standing of the Sisters. I know that they own a place on South Division and Michigan streets. If they must build, why not build where they are now? The main object is, this little park is overcrowded on nice summer nights, especially when there is a concert; more than 10,000 are inside and outside on the street. How could any wagon call get through such crowds without hurting or killing several people, which will be the case if the same is erected.

M. STRAUSS.

Mr. Strauss's original objection was that it would be unpleasant for visitors to be associated with convalescents. Now he has another cause of complaint. Up to the present writing, however, there have been no changes in the plans of the Sisters of Charity regarding their new hospital. It will go up.

